# Case Report: zanidatamab-based regimen as a potential later-line therapeutic option in HER2-amplified metastatic colorectal cancer

**DOI:** 10.3389/fphar.2026.1925069

**Published:** 2026-09-21

**Authors:** Lin Meng, Wanqian Zhang, Chaoqun Sun, Xiujuan Qu, Ying Chen

**Affiliations:** 1 Department of Medical Oncology, The First Hospital of China Medical University, Shenyang, China; 2 Key Laboratory of Anticancer Drugs and Biotherapy of Liaoning Province, The First Hospital of China Medical University, Shenyang, China; 3 Liaoning Province Clinical Research Center for Cancer, The First Hospital of China Medical University, Shenyang, China; 4 Key Laboratory of Precision Diagnosis and Treatment of Gastrointestinal Tumors, Ministry of Education, The First Hospital of China Medical University, Shenyang, China

**Keywords:** case report, HER2 positive, late-line treatment, metastatic colorectal cancer (mCRC), zanidatamab

## Abstract

**Background:**

Overexpression human epidermal growth factor receptor 2 (HER-2) has been identified as a key oncogenic driver in metastatic colorectal cancer (mCRC), detected in between 2% and 5% of the overall mCRC population, with a higher prevalence identified in RAS/RAF/PI3KCA wild-type patients. Optimal targeted interventions are seldom administered to patients with HER2-positive mCRC due to the absence of definitive anti-HER2 therapeutic regimens in the refractory setting. As a result, for patients who have undergone multiple lines of therapy—particularly those with cetuximab resistance driven by ERBB2 amplification-mediated bypass activation—treatment strategies are frequently confined to best palliative care or participation in clinical trials.

**Case presentation:**

This report demonstrates a case of multi-line refractory mCRC with high-level HER2 overexpression. Following multi-line treatment therapies, the patient was treated with a zanidatamab-based chemotherapy salvage regimen, leading to a partial response (PR) and an ongoing progression-free survival of approximately 10 months.

**Conclusion:**

This case study highlights that the combination of zanidatamab with liposomal irinotecan and raltitrexed may provide an encouraging anti-tumor therapeutic option for mCRC patients with extremely high-level HER-2 overexpression and resistance to conventional anti-EGFR therapies. These findings offer valuable clinical insights into combining bispecific anti-HER2 targeted therapy with an optimized chemotherapy backbone, warranting further evaluation in prospective trials. Nevertheless, future prospective trials with larger cohorts are warranted to establish its survival benefits and universal applicability definitively.

## Introduction

Colorectal cancer (CRC) ranks among the top-three most frequently diagnosed malignancies worldwide and accounts for the second highest number of cancer-related deaths globally. Based on global cancer statistics 2022, CRC accounted for approximately 1.9 million new cases (9.6% of all cancer diagnoses) and led to more than 900,000 deaths (9.3% of all cancer-related deaths) (Bray et al., 2024). Despite improvements in early screening and multidisciplinary care, nearly 20% of patients initially present with metastasis, and the 5-year survival for advanced disease remains poor at roughly 14% (Shin et al., 2023). Molecular characterization via IHC, fluorescence *in situ* hybridization, and next-generation sequencing (NGS), used to identify actionable targets, now serves as the foundation of advanced treatment, thereby facilitating the delivery of tailored targeted therapies based on individual molecular profiles to enhance mCRC survival (Steup et al., 2025).

Among a multitude of emerging biomarkers, amplification of the presence of human epidermal growth factor receptor 2 (HER2) represents an increasingly pivotal oncogenic driver. Aberrant amplification and overexpression of HER2 facilitate ligand-independent receptor dimerization, which potently activates the downstream RAS-MAPK and PI3K/AKT signaling pathways, thereby endowing tumor cells with malignant proliferative and anti-apoptotic advantages (Raghav and Moasser, 2023). Thus far, HER2-targeted therapies have become the cornerstone of frontline systemic therapy for patients with HER2-positive breast, gastric, and gastroesophageal junction cancers (Gradishar et al., 2024; Ajani et al., 2025). Despite this, there are currently no globally approved first-line targeted therapies for HER2-positive mCRC, strictly limiting the alternatives available to advanced-stage patients. While accumulating evidence from exploratory trials has elucidated the potential role of antibody-drug conjugates (ADCs) and tyrosine kinase inhibitors (TKIs) (Sartore-Bianchi et al., 2016), the overall efficacy has proven to be limited. To address these limitations, zanidatamab—a novel biparatopic HER2 bispecific antibody—simultaneously engages non-overlapping ECD2 and ECD4 domains, thereby accelerating receptor internalization and degradation (Weisser et al., 2023). Already approved for previously treated HER2-positive biliary tract cancer, zanidatamab has demonstrated encouraging activity beyond this initial indication. At the 2024 European Society for Medical Oncology Annual Meeting, investigators reported outcomes from a study of zanidatamab combined with FOLFOX chemotherapy as an upfront therapeutic strategy in HER2-positive mCRC. Notably, among 11 response-evaluable patients followed for a median of 15.4 months, an impressive objective response rate (ORR) of 90.9% was observed, and no patient experienced disease progression, yielding a disease control rate (DCR) of 100%. Our study presents the case of a multiline-refractory mCRC patient characterized by a RAS/BRAF wild-type profile and extremely high-level HER2 overexpression. Facing a refractory setting following successive resistance to trastuzumab, HER2-targeted ADCs, and cetuximab, the patient received a late-line salvage therapy centered on the combination of zanidatamab with liposomal irinotecan and raltitrexed. Remarkably, this regimen has achieved a clinical benefit of 9 months at time of writing.

## Case representation

A 43-year-old female who initially presented with hematochezia was subsequently admitted to our hospital in 2021. Her colonoscopy revealed an ill-defined, ulcerated, purplish-red mass located 100 mm from the anal verge, with an eroded, friable, surface. Concurrent PET-CT indicated multiple pulmonary metastases and multiple enlarged lymph nodes around the rectum. She received a radical anterior resection for rectal cancer on 16 June 2021, TNM stage was pT4apN1acM1a. Her resected specimen’s pathological analysis established a diagnosis of rectal adenocarcinoma, with immunohistochemical staining revealing the following results: a proficient mismatch repair (pMMR/MSS) phenotype, C-erbB-2 (positive, 3+); the NGS results of the tumor tissue are as follows: ERBB2 amplification (CN: 48.3) (Table 1); KRAS/NRAS/BRAF wild-type; heterozygous UGT1A1 polymorphism; and programmed-death ligand 1 (PD-L1) (CPS = 5). Considering these results, we made a diagnosis of HER2-positive metastatic rectal cancer with lung metastases. Baseline laboratory evaluations, including complete blood counts and liver and renal function panels were unremarkable. The patient had no foundational comorbidities, no personal or family history of cancer, and an ECOG performance status of 1. Considering the potentially limited benefit of anti-EGFR monoclonal antibody therapy for HER2-positive CRC, first-line treatment combining trastuzumab with the CapeOX regimen was initiated on 7 September 2021. After completion of seven cycles, maintenance therapy with trastuzumab and capecitabine was administered. The best therapeutic evaluation was a PR. The primary adverse effects observed were Grade-2 neurotoxicity and Grade-2 myelosuppression, with a first-line time to progression (TTP) of 16 months.

**TABLE 1:** Immunohistochemical (IHC) and Next-generation sequencing (NGS) results of tissue samples and plasma.HER2 protein status was strictly interpreted using the CRC-specific HERACLES diagnostic criteria (IHC 3+ defined as strong membranous reactivity in≥ 50% of tumor cells).

Detection time	Biopsy source	HER2 status	
		IHC	NGS(ERBB2 amplification)
June 27, 2021	Rectum	3+	CN:48.3
	Blood	—	Negative
July 15, 2024	Blood	—	Negative
May 30, 2025	Lung	3+	CN:41.41
	Blood	—	Negative

IHC Technical Details:Primary rabbit monoclonal anti-HER2/neu antibody (Clone 4B5, Ventana Medical Systems, Tucson, AZ, USA) performed on an automated BenchMark ULTRA stainer.

NGS Technical Details: Targeted next-generation sequencing was performed using the GeneseeqPrime® panel (Geneseeq Technology Inc., Nanjing, China). Based on the manufacturer’s validated algorithm, *ERBB2* gene amplification was defined as a gene copy number (CN) threshold of ≥4.0 for FFPE tissue samples and≥3.2 for plasma ctDNA samples; copy numbers below these cutoffs were classified as negative.

Subsequently, on 9 January 2023, a second-line therapy comprising the mXELIRI regimen combined with bevacizumab was administered. Following seven cycles thereof, the patient received maintenance therapy with bevacizumab and capecitabine. The best objective response was stable disease, with Grade-2 nausea and vomiting as the principal adverse reactions. The second-line TTP reached 7 months.

On 7 September 2023, the patient was enrolled in a third-line treatment as part of a real-world, prospective, multicenter, single-arm observational study evaluating TAS-102 in combination with regorafenib or fruquintinib for the treatment of advanced CRC in the third-line (or beyond) setting, and received the regimen of regorafenib plus TAS-102. This therapeutic approach achieved a best response of SD. The predominant adverse effects were Grade-2 fatigue and Grade-2 proteinuria, with a maximum 24-h urinary protein quantification of 1.5 g. The TTP for the third-line therapy was 11 months.

Before fourth-line therapy, liquid biopsy using next-generation sequencing technology was subsequently conducted on a blood sample collected from the patient, blood NGS revealed a KRAS/NRAS/BRAF wild-type profile with no ERBB2 amplification. On 12 August 2024, the patient participated in a Phase-I trial for TQB2102 (a HER2-targeted agent), achieving stable disease with a 6-month TTP. Primary adverse events included Grade-3 leukopenia and thrombocytopenia. Given the consistent KRAS/NRAS/BRAF wild-type status in both initial tissue and post-treatment blood, fifth-line cetuximab plus CapeOX was initiated on 25 March 2025, however, disease progression occurred after only three cycles (TTP: 2.5 months).

A subsequent lung biopsy confirmed metastatic colorectal adenocarcinoma with HER2 3+ and persistent ERBB2 amplification (NGS CN: 41.41) (Table 1). Given that zanidatamab has been approved for the second-line treatment of HER2-positive biliary tract cancer, a sixth-line salvage regimen that combined zanidatamab (1,200 mg), liposomal irinotecan (61.19 mg), and raltitrexed (3 mg) was initiated on 4 June 2025. Given her diagnosis of early-onset colorectal cancer (EOCRC) at age 43, comprehensive hereditary cancer screening was conducted. Germline multigene panel testing revealed no pathogenic germline variants associated with Lynch syndrome or other polyposis syndromes, and tumor tissue demonstrated a proficient mismatch repair (pMMR/MSS) phenotype. Pharmacogenomic testing identified a heterozygous UGT1A1 polymorphism. Because UGT1A1 intermediate metabolizer status can impair SN-38 clearance and heighten the risk of irinotecan-induced severe diarrhea and neutropenia (Gammal et al., 2016), liposomal irinotecan dosing was carefully calibrated (61.19 mg). After three cycles, the patient achieved a PR with an ongoing TTP of approximately 10 months (Figure 1). Before each treatment cycle, standard premedication consisting of intravenous dexamethasone (10 mg) and Promethazine (25 mg) were routinely administered to prevent chemotherapy-induced nausea, vomiting and infusion-related reactions. Following the second treatment cycle, the patient experienced Grade 2 diarrhea, representing a known overlapping gastrointestinal toxicity of zanidatamab, liposomal irinotecan, and raltitrexed. Prompt supportive care with oral loperamide (4 mg loading dose followed by 2 mg every 2 h) and oral rehydration therapy led to complete resolution of symptoms within 48 h. No severediarrhea, neutropenia, or other treatment-emergent adverse events occurred. The patient now receives a regular treatment at our institution and enjoys a good quality of life (Figure 2).

**FIGURE 1:**
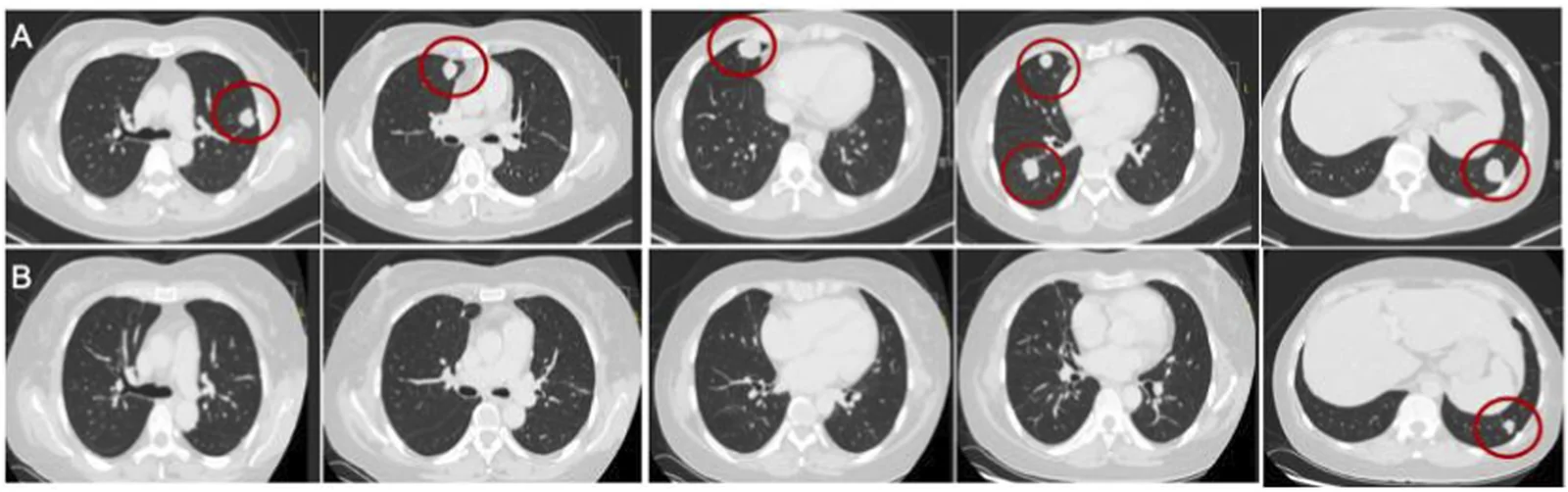
Variations of pulmonary metastatic lesions shown by chest CT scans before and after six-line treatment (red arrow). **(A)** Observed on 30 May 2025; **(B)** Observed on 14 March 2026. Scale bar on the right side indicates 10 cm.

**FIGURE 2:**
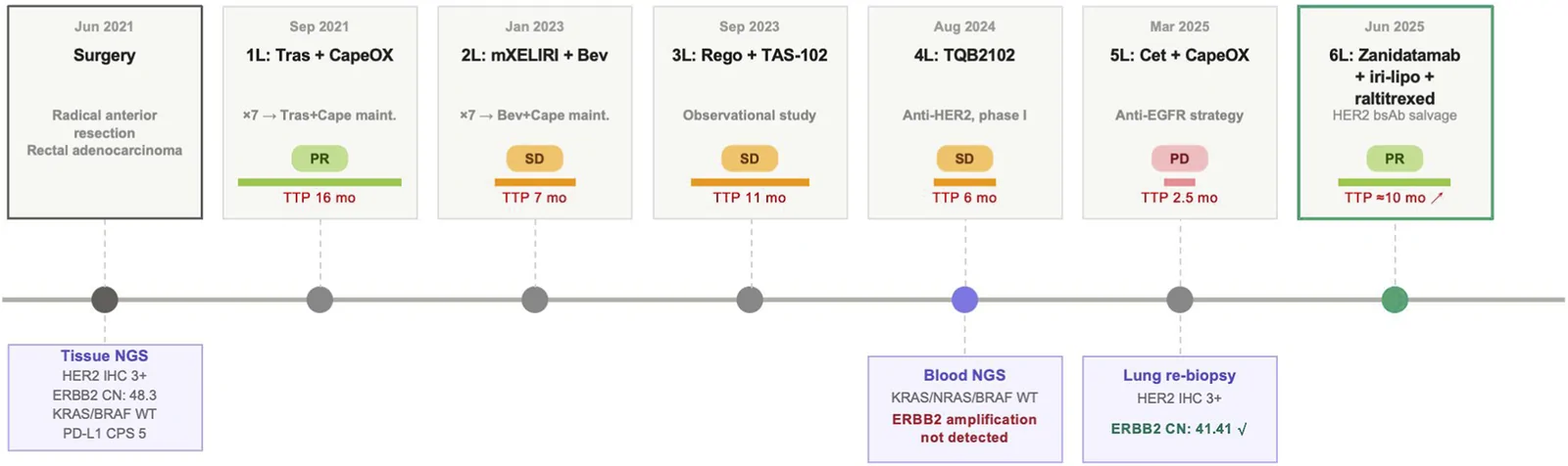
Clinical timeline of sequential therapies for HER2-amplified metastatic rectal cancer.

## Discussion

HER2 positivity accounts for 2%–5% of all CRC, but its incidence increases to more than 5%–10% in the RAS/BRAF wild-type mCRC subgroup (Strickler et al., 2023). China currently lacks approved HER2-targeted therapies for CRC, emerging clinical trials confirm the survival benefits of anti-HER2 agents. In the DESTINY-CRC01 trial, T-DXd (DS-8201) demonstrated robust efficacy in 53 patients with KRAS wild-type, HER2 positive mCRC refractory to at least two prior standard systemic therapies, achieving an ORR of 45.3%, mPFS of 6.9 months, and mOS of 15.5 months (Siena et al., 2021).

Strikingly, patients previously treated with anti-HER2 agents retained an ORR of 43.8%. Meanwhile, the MOUNTAINEER trial evaluated tucatinib plus trastuzumab in chemotherapy-refractory, HER2-positive, RAS wild-type mCRC, yielding an ORR of 39.3%, a 15.2-month DOR, an 8.1-month mPFS, and a 23.9-month mOS (Strickler et al., 2026). Despite small cohorts, these studies establish anti-HER2 therapy as a crucial salvage option. At time of writing, the MOUNTAINEER-03 trial is ongoing to evaluate the frontline potential of combining tucatinib, trastuzumab, and chemotherapy (Strickler et al., 2025).

Notably, the success of the sixth-line salvage regimen in this case was ascribed to the introduction of zanidatamab. Zanidatamab is a next-generation biparatopic HER2-targeted bispecific antibody that engages two non-overlapping epitopes—the juxtamembrane domain (ECD4) and the dimerization domain (ECD2)—simultaneously and with dual specificity. This unique binding mechanism promotes receptor clustering, rapid internalization, and potent downregulation of cell-surface HER2, while inducing robust antibody TTP-dependent cellular cytotoxicity (Weisser et al., 2023). In the landmark, global, multicenter, Phase-IIb HERIZON-BTC-01 trial, zanidatamab monotherapy showed a remarkable objective response rate (ORR) of 41.3% and a median duration of response of 12.9 months in patients with HER2-amplified advanced biliary tract cancer (BTC) who had failed prior standard first-line chemotherapy (Harding et al., 2023). This robust evidence base not only led to the FDA’s late-2024 approval of zanidatamab for previously treated HER2-positive biliary tract cancer, but also served as a strong clinical rationale for its use in our multiline-refractory, extreme HER2-amplified mCRC case. A prospective, multicenter, Phase-I trial (Meric-Bernstam et al., 2022) highlighted the remarkable efficacy of zanidatamab monotherapy in HER2-amplified/expressing mCRC, with a 38% ORR, 77% DCR, and 6.8-month mPFS. More importantly, it proved capable of inducing tumor shrinkage even in patients refractory to prior HER2-targeted treatment (Meric-Bernstam et al., 2022). This finding is consistent with the remarkable clinical benefit observed in our patient, having achieved a PR and a PFS benefit of approximately 10 months through a zanidatamab-based combination.

While prior clinical trials have demonstrated the efficacy of zanidatamab monotherapy and zanidatamab plus mFOLFOX6, the clinical selection of liposomal irinotecan and raltitrexed as the cytotoxic backbone alongside zanidatamab in this patient was based on specific therapeutic considerations (Kalra et al., 2014; Cheng et al., 2024). Switching to liposomal irinotecan—which offers prolonged plasma half-life and enhanced tumor-specific deposition compared to conventional irinotecan—combined with raltitrexed, a selective thymidylate synthase inhibitor, provided a potent, promising salvage strategy. Mechanistically, anti-HER2 targeted therapies and cytotoxic agents act synergistically: bispecific binding of zanidatamab blocks receptor dimerization and downstream survival signaling, rendering tumor cells more sensitive to chemotherapy-induced DNA damage and apoptosis (Raghav and Moasser, 2023). In addition, no standard treatment regimen is currently recommended by guidelines for later-line patients. From a safety standpoint, overlapping gastrointestinal toxicities—particularly diarrhea—warrant strict vigilance when combining zanidatamab with liposomal irinotecan and raltitrexed. Diarrhea occurs in 43%–52% of patients receiving zanidatamab monotherapy (Meric-Bernstam et al., 2022) and remains a key dose-limiting toxicity for irinotecan. Although our patient experienced only Grade 2 diarrhea, which promptly resolved with loperamide, proactive surveillance and early supportive care are vital to prevent severe adverse events with this combination.

This case provides valuable insights into treatment selection for RAS wild-type, HER2-positive advanced CRC. While a RAS wild-type profile typically indicates anti-EGFR efficacy, our patient progressed on fifth-line cetuximab after merely 2.5 months. This rapid failure, contrasting starkly with their durable anti-HER2 responses, is consistent with high-level ERBB2 amplification as a primary driver of intrinsic anti-EGFR resistance (Zhou et al., 2021). Mechanistically, extreme ERBB2 amplification promotes highly active HER2/HER3 heterodimerization, which circumvents anti-EGFR blockade via compensatory PI3K/AKT pathway activation (Yonesaka et al., 2011). Therefore, prioritizing anti-HER2 over anti-EGFR therapies warrants careful consideration in ERBB2-amplified mCRC, even with a RAS wild-type status.

In recent years, the spatio-temporal heterogeneity of HER2 in mCRC has been identified as a critical mechanism underlying the resistance to traditional anti-HER2 targeted therapeutic regimens (Siravegna et al., 2018). Notably, despite the selective pressure of multiple systemic and anti-EGFR regimens, strong HER2 overexpression was persistently maintained in the lung metastatic tissue, even though plasma ctDNA testing did not detect ERBB2 amplification. Concordance between tissue and liquid biopsy in HER2-positive CRC is known to be incomplete; recent analysis from the MyPathway trial revealed that approximately 31% of tissue-confirmed HER2-positive CRC cases failed to show ERBB2 amplification on ctDNA, primarily due to variations in ctDNA shedding and tumor burden (Meric-Bernstam et al., 2025). Thus, negative ctDNA does not preclude tissue HER2 overexpression. This finding emphasizes that such patients can derive continuous benefit from sustained anti-HER2 regimens, thus avoiding clinical delays associated with ineffective alternatives.

In conclusion, this case provides promising clinical evidence supporting the efficacy of zanidatamab combined with liposomal irinotecan and raltitrexed in a heavily pretreated mCRC patient with persistent high-level ERBB2 amplification. Despite the encouraging outcomes observed in this case report, we must acknowledge certain limitations therein. Inherently, a single retrospective case report offers a limited level of evidence. Therefore, the definitive efficacy of zanidatamab-based combination regimens in mCRC requires further validation in large-scale prospective trials. Moreover, the long-term safety monitoring and adverse events necessitate continuous clinical follow-up and in-depth mechanistic investigation.

## Data Availability

The original contributions presented in the study are included in the article/supplementary material, further inquiries can be directed to the corresponding authors.
